# An Atypical Cause of Upper Gastrointestinal Bleeding in an Elderly Patient

**DOI:** 10.7759/cureus.100156

**Published:** 2025-12-26

**Authors:** Hari Movva, Devin Garza, Minh Tran, Jing He, Sreeram Parupudi

**Affiliations:** 1 Internal Medicine, University of Texas Medical Branch at Galveston, Galveston, USA; 2 School of Medicine, University of Texas Medical Branch at Galveston, Galveston, USA; 3 Gastroenterology and Hepatology, University of Texas Medical Branch at Galveston, Galveston, USA; 4 Pathology, University of Texas Medical Branch at Galveston, Galveston, USA

**Keywords:** elderly population, gastric hyperplastic polyp, iron deficiency anemia, lower gi bleed, melena

## Abstract

Hyperplastic gastric polyps are typically benign and asymptomatic, often found incidentally during endoscopy. However, rare cases may present with bleeding, especially when polyps are large, ulcerated, or pedunculated. We report a 69-year-old male with melena and symptomatic anemia who was found to have a 3.6 cm ulcerated, pedunculated gastric polyp on endoscopy. The lesion was resected with a snare, and hemostasis was achieved with hemoclips. Pathology confirmed a hyperplastic polyp without dysplasia or malignancy. This case highlights the importance of considering bleeding polyps in the differential diagnosis of upper gastrointestinal (GI) bleeding and the dual diagnostic-therapeutic role of endoscopic resection.

## Introduction

Gastric polyps are typically incidental findings during upper gastrointestinal endoscopy performed for unrelated indications. In most cases, they are asymptomatic; however, some polyps may cause symptoms and carry malignant potential, warranting appropriate evaluation and management. Gastric polyps are identified in approximately 6% of upper gastrointestinal endoscopies in the United States [1].

Common types of gastric polyps include hyperplastic polyps (more frequent in regions with high *Helicobacter pylori* prevalence), fundic gland polyps (more common in Western populations, often linked to proton pump inhibitor (PPI) use), adenomas, neuroendocrine tumors, and inflammatory fibroid polyps [1]. While most gastric polyps are benign and asymptomatic, they can occasionally result in complications such as abdominal pain, bleeding, anemia, and even malignancy [2,3].

Hyperplastic polyps represent 30% to 93% of all gastric polyps detected on endoscopy, particularly in the antrum, and are frequently associated with *H. pylori* infection [2,3]. Though bleeding from gastric hyperplastic polyps is rare, it can be a significant cause of gastrointestinal blood loss, particularly in older adults [2]. Haddad et al. reported that 1.4% of upper gastrointestinal bleeding cases were attributed to hyperplastic polyps in the gastric antrum [2].

## Case presentation

A 69-year-old male with a past medical history of hypertension and hyperlipidemia presented to the emergency department with four to five days of progressive dizziness and near-syncope. He reported lightheadedness that worsened with standing and exertion, along with generalized fatigue and mild shortness of breath. He denied chest pain, palpitations, nausea, vomiting, hematemesis, hematochezia, lower extremity swelling, visual disturbances, or focal neurological symptoms. He had no prior history of gastrointestinal bleeding, NSAID (nonsteroidal anti-inflammatory drugs) use, or anticoagulation therapy. Notably, he had never undergone an endoscopic evaluation and had not had a colonoscopy in over 10 years, despite a family history of colon cancer in his grandmother. He had a significant remote smoking history of 84 pack-years and quit alcohol approximately 1.5 years prior.

On physical examination, the patient was hemodynamically stable. However, initial laboratory evaluation revealed severe anemia with a hemoglobin level of 3.5 g/dL (Table 1), down from 13.0 g/dL one year earlier. Additional abnormalities included leukocytosis (white blood cell 29.58 × 10³/μL), mild hyponatremia (Na 133 mmol/L), and a slightly elevated creatinine level (1.30 mg/dL). Platelet count and troponin were within normal limits. Digital rectal examination confirmed the presence of melena. Computed tomography of the abdomen and pelvis with intravenous contrast demonstrated mildly thickened gastric walls and submucosal edema, findings suggestive of gastritis. Hyperdense material within the stomach was consistent with either ingested contents or intraluminal blood.

**Table 1:** Laboratory investigations on admission in the emergency department

Laboratory Test	Value	Reference Range
White blood cell count (WBC)	29.58 × 10³/μL	4.22–10.7 x10³/uL
Hemoglobin (Hgb)	3.5 g/dL	12.2–16.4 g/dL
Platelet count (PLT)	261 x 10³/μL	150–328 x10³/uL
Sodium (Na)	133 mmol/L	135–145 mmol/L
Creatinine (Cr)	1.30 mg/dL	0.60–1.25 mg/dL
Troponin (Trop)	0.005 ng/mL	≤0.034 ng/mL
Magnesium (Mg)	2.1 mg/dL	1.7-2.4 mg/dL

**Figure 1:**
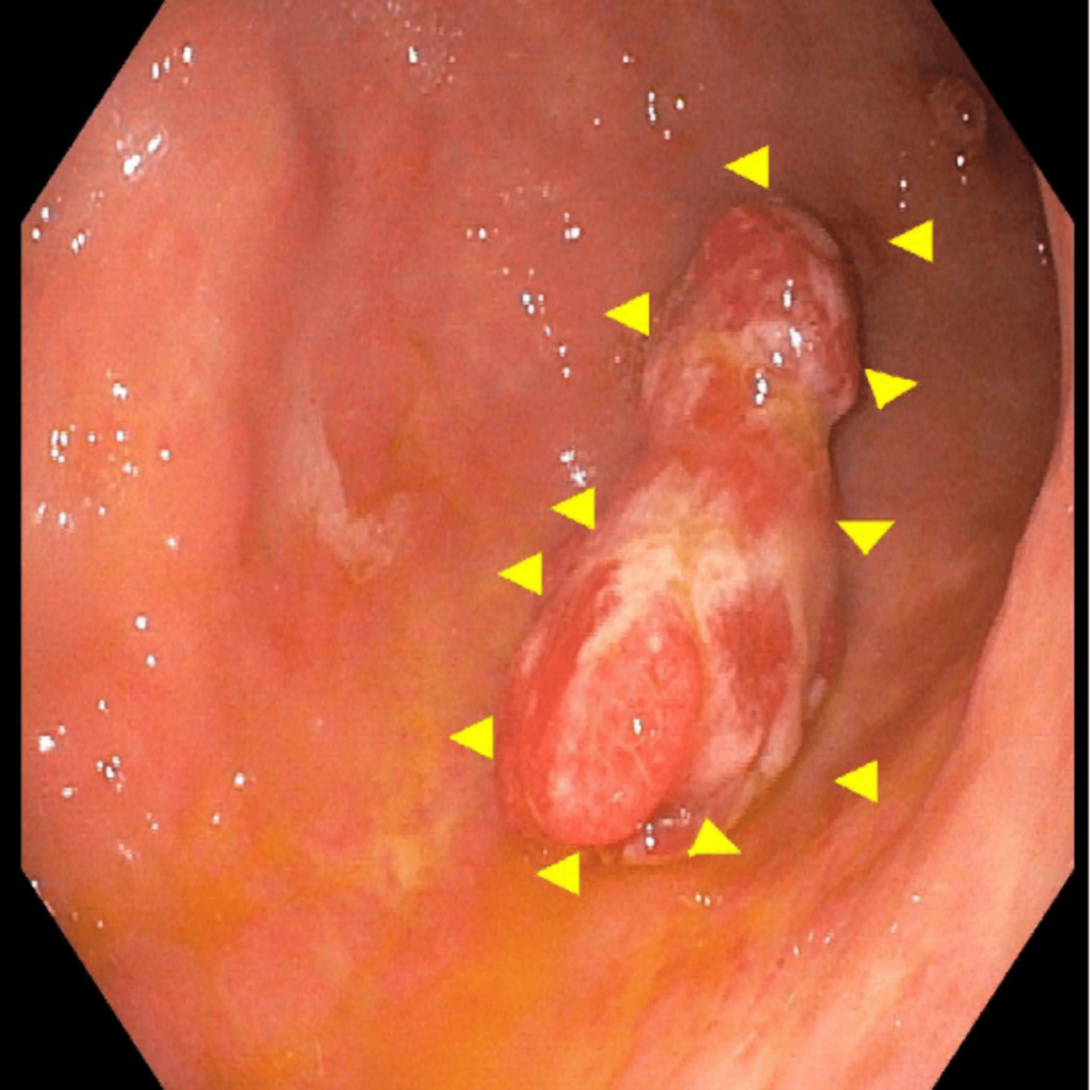
Pedunculated hyperplastic polyp found in the gastric body under white light White light endoscopy revealed a large, pedunculated polyp located in the gastric body. The lesion exhibited a lobulated contour and prominent stalk, consistent with a hyperplastic polyp. The surface appeared irregular and erythematous, with evidence of mucosal ulceration. Arrows delineate the margins of the polyp.

Esophagogastroduodenoscopy (EGD) revealed a large, pedunculated polyp in the gastric body with a lobulated contour and an ulcerated surface, actively oozing blood, findings that strongly suggested it as the source of the patient’s upper gastrointestinal bleeding. The size, morphology, and ulcerated surface of the polyp raised concern for chronic blood loss contributing to the patient’s severe iron deficiency anemia. Under white light endoscopy, the lesion was clearly delineated, with arrows marking its margins for documentation (Figure 1).

**Figure 2:**
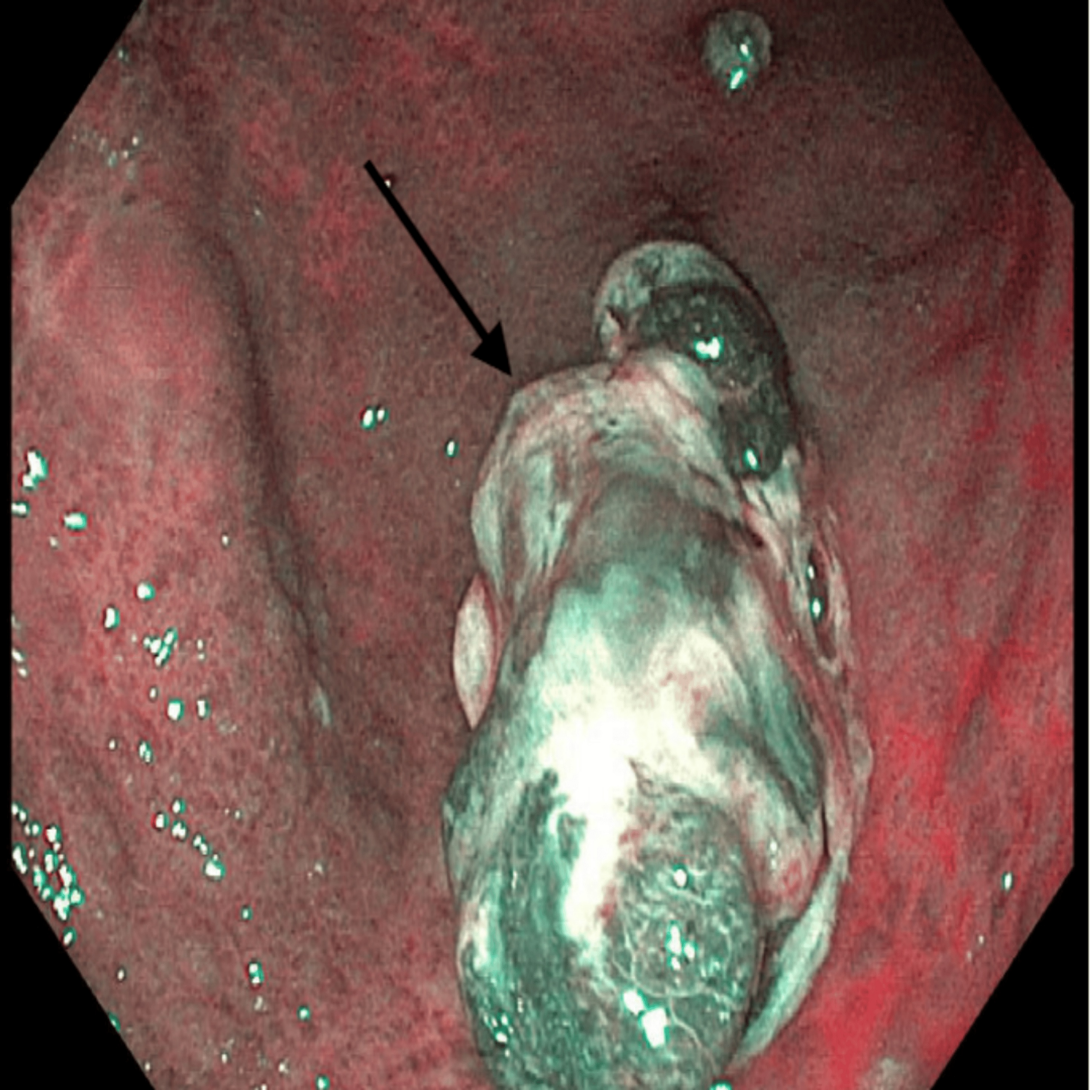
Pedunculated hyperplastic polyp found in the gastric body under narrow-band imaging Narrow-band imaging (NBI) endoscopy of the gastric body demonstrates a large, pedunculated hyperplastic polyp as delineated by the arrow. In this image, the polyp's lobulated surface and vascular structures are more clearly visualized compared to white light. The absence of irregular or disrupted microvascular patterns and the presence of a regular pit pattern were consistent with a benign hyperplastic lesion.

To further evaluate the lesion, narrow-band imaging (NBI) was employed to emphasize mucosal and vascular patterns, which aids in identifying subtle features not visible with conventional white light. NBI improved characterization of the polyp’s surface pit pattern and microvascular architecture, helping to assess for dysplasia or early malignant transformation (Figure 2). The absence of irregular or disorganized vascular patterns made a malignant process less likely, but definitive diagnosis still required histologic evaluation.

**Figure 3:**
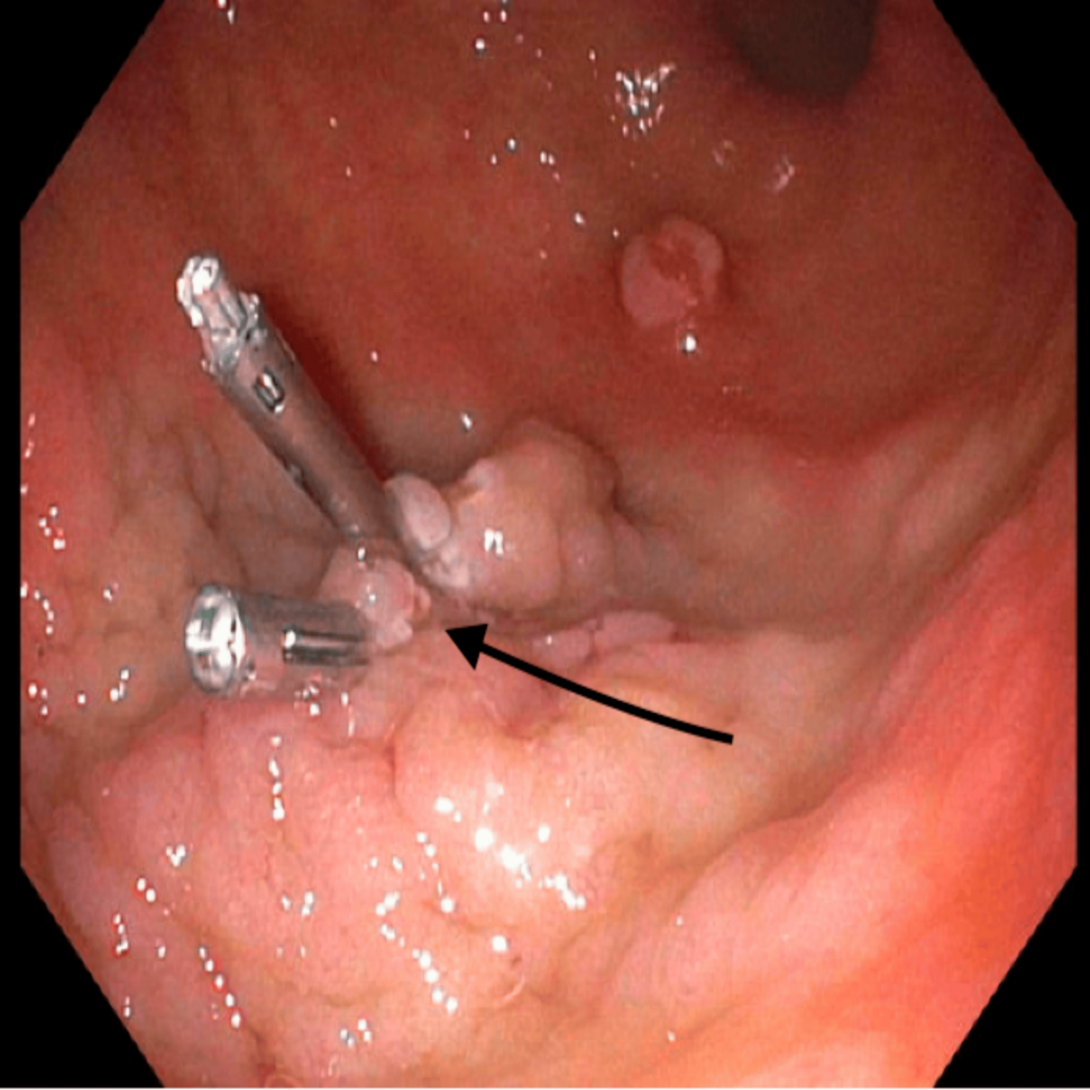
Gastric body status post resection with hemoclips closing the defect Endoscopic view of the gastric body following resection of a large, pedunculated hyperplastic polyp. The polypectomy site demonstrates a clean mucosal defect, which was successfully closed using hemoclips to ensure hemostasis and reduce the risk of post-procedural bleeding as delineated by the arrow. The post-polypectomy site showed no evidence of immediate complications, and clip placement was technically successful with adequate closure of the mucosal defect.

Due to ongoing slow bleeding and the lesion’s high-risk features, which consisted of size >2 cm, pedunculated morphology, and mucosal ulceration, endoscopic snare polypectomy was performed to both treat the source of bleeding and obtain tissue for histopathologic diagnosis. Complete removal of large polyps is essential not only to halt bleeding but also to assess for dysplasia or malignancy. After resection, hemoclips were deployed to close the mucosal defect and secure hemostasis, minimizing the risk of post-polypectomy bleeding (Figure 3).

**Figure 4:**
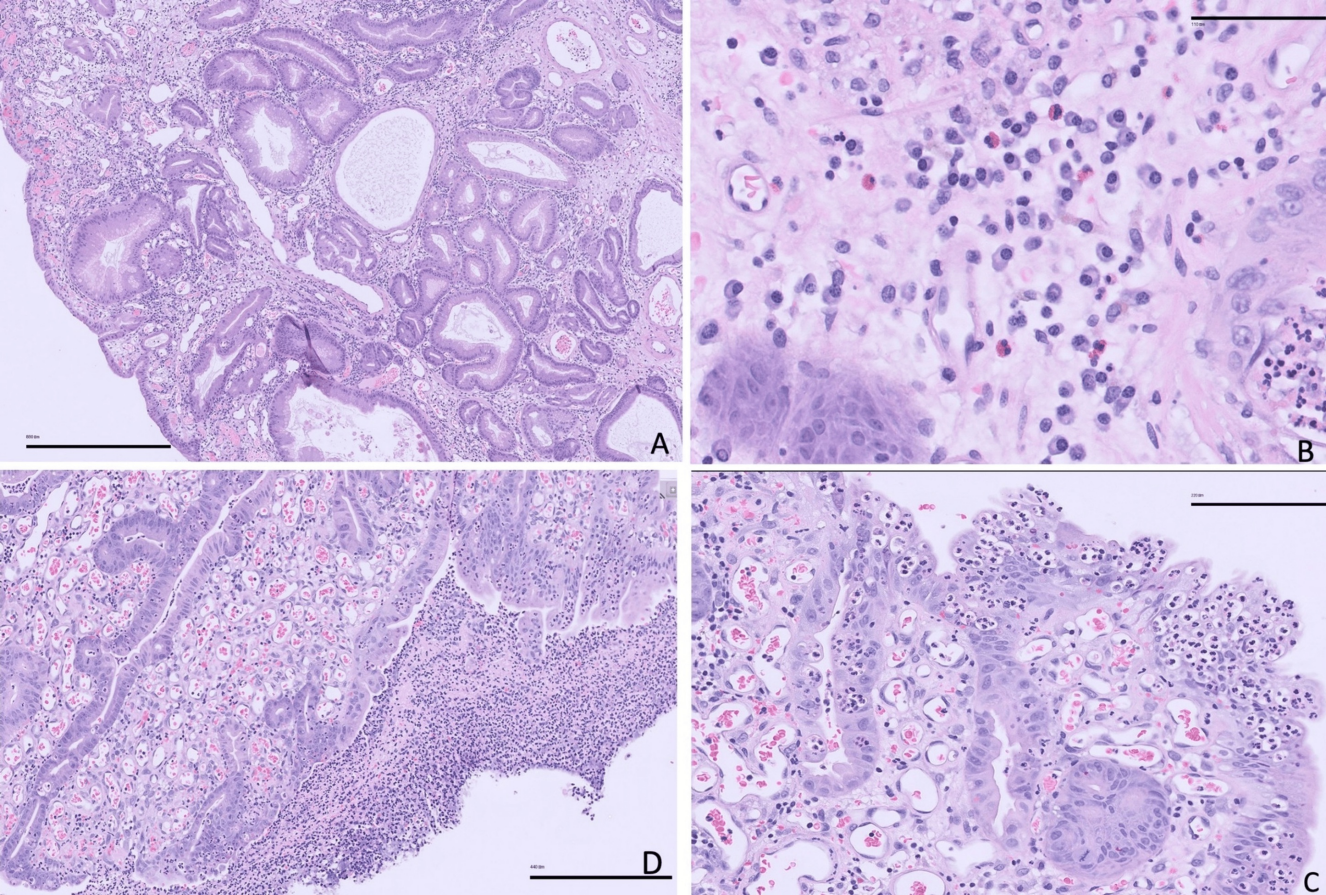
Histological findings of the gastric polyp (A) The foveolar epithelium demonstrates elongation, architectural distortion, and irregular shape with cystic dilatations (50x). (B) The lamina propria of this polyp shows edema, congestion, and acute and chronic inflammation (400x). (C) Mucosal erosion areas show intraepithelial neutrophils and granulation tissue (200x). (D) Focal mucosal ulceration (100x).

Histopathological analysis of the 3.6 cm endoscopically resected gastric lesion confirmed a hyperplastic polyp characterized by surface ulceration and reactive epithelial changes. Notably, there was no evidence of intestinal metaplasia, adenomatous transformation, dysplasia, or Helicobacter pylori infection (Figure 4). These features support the diagnosis of a benign but clinically significant polyp contributing to chronic gastrointestinal blood loss.

Microscopically, the foveolar epithelium demonstrated elongation, architectural distortion, and cystic dilatation (Figure 4A, 50×), findings typical of hyperplastic polyps and indicative of regenerative changes in response to chronic mucosal injury. The lamina propria showed marked edema, congestion, and a mixed inflammatory infiltrate composed of both acute and chronic inflammatory cells (Figure 4B, 400×), consistent with ongoing inflammation. Areas of mucosal erosion were associated with intraepithelial neutrophil infiltration and granulation tissue formation (Figure 4C, 200×), while focal mucosal ulceration was also observed (Figure 4D, 100×). These histologic changes correlate with the clinical presentation of chronic gastrointestinal bleeding and support the necessity of polyp removal.

The patient received multiple units of packed red blood cells, resulting in an improvement of hemoglobin to 7.3 g/dL. He remained clinically stable throughout his hospitalization and experienced no further episodes of gastrointestinal bleeding. He was discharged in stable condition with scheduled outpatient follow-up.

## Discussion

We present a case of severe iron deficiency anemia in an elderly patient caused by chronic gastrointestinal blood loss secondary to a large hyperplastic gastric polyp. While hyperplastic polyps are commonly regarded as benign, smooth, dome-shaped lesions typically measuring 0.5-1.5 cm in diameter, they can occasionally grow larger, become lobulated or pedunculated, and develop ulcerations on their surface that predispose to bleeding [4,5]. In such instances, their clinical significance becomes more pronounced, particularly due to the potential for chronic occult blood loss and the rare but notable risk of neoplastic transformation.

Hyperplastic polyps account for approximately 30-93% of all gastric polyps identified on upper endoscopy, depending on the population studied and regional prevalence of *Helicobacter pylori* infection [6,7]. Although they are typically considered non-neoplastic, evidence suggests that dysplastic changes or even malignant transformation can occur, particularly in larger or atypical polyps. Histological studies have demonstrated dysplasia or carcinoma in 5%-19% of resected hyperplastic polyps [8,9], a finding that underpins current endoscopic management recommendations.

The American Society for Gastrointestinal Endoscopy (ASGE) recommends removal of gastric polyps ≥0.5 cm via endoscopic polypectomy, particularly when symptomatic, large, pedunculated, or ulcerated, due to their malignant potential and risk of ongoing blood loss [10]. This recommendation aligns with several observational studies reporting increased rates of dysplasia in larger polyps and those with surface erosions [11,12].

In a nationwide U.S. study involving over 120,000 esophagogastroduodenoscopies (EGDs), 1,127 biopsy-proven hyperplastic polyps were identified. Among these, five exhibited low-grade dysplasia, although no cases of high-grade dysplasia or carcinoma were reported [1]. While these data support the generally benign nature of these lesions, they also reinforce the need for vigilance, particularly in symptomatic patients or those with atypical polyp morphology.

In the present case, endoscopic resection was pursued due to the polyp’s size (>2 cm), pedunculated configuration, and ulcerated surface, which were consistent with chronic blood loss as the source of the patient’s iron deficiency anemia. The pathology revealed a hyperplastic polyp with surface erosion but no intestinal metaplasia, dysplasia, or evidence of *H. pylori* infection. This is consistent with previous case reports documenting large hyperplastic polyps causing iron deficiency anemia via chronic mucosal bleeding in the absence of overt malignancy [13,14].

Eradication of *H. pylori* is also an essential component of managing hyperplastic polyps. Multiple studies have demonstrated that clearance of *H. pylori* can lead to regression or complete disappearance of hyperplastic polyps, especially those smaller than 1 cm [15-18]. Even in cases where the polyps are resected, testing for and treating *H. pylori* is recommended as a preventive strategy against recurrence and further gastric mucosal injury.

Given the potential for insidious clinical manifestations, such as iron deficiency anemia without overt gastrointestinal bleeding, endoscopists should maintain a high index of suspicion when evaluating large or atypical gastric polyps. In addition to treating acute or chronic blood loss, histopathologic examination remains crucial to rule out dysplasia or carcinoma and to guide surveillance and management.

## Conclusions

Hyperplastic gastric polyps are typically asymptomatic and incidentally discovered during upper endoscopy. However, they may occasionally present with iron deficiency anemia due to chronic blood loss, particularly when large or ulcerated. Ultimately, this case reinforces the need for vigilance when encountering large or ulcerated gastric polyps. Prompt recognition, endoscopic intervention, and microbiologic evaluation for *H. pylori* not only mitigate the risk of malignancy and recurrent bleeding but also contribute to improved patient outcomes. Increased awareness of these clinical nuances can aid in early detection, appropriate intervention, and long-term surveillance strategies for at-risk patients.
